# Ectopic Expression of *AsDof6.5* from Oat (*Avena sativa* L.) Enhances Low Nitrogen Tolerance in Transgenic *Arabidopsis thaliana*

**DOI:** 10.3390/genes17101248

**Published:** 2026-10-09

**Authors:** Yue Wang, Yincui Zhang, Kaiqiang Liu, Qingping Zhou

**Affiliations:** 1 School of Ecology and Environmental Science, Qinghai Institution of Technology, Xining 810016, China; joanwy919@163.com (Y.W.); yinczhang@qhit.edu.cn (Y.Z.); 2 Qinghai Academy of Animal Science and Veterinary Medicine, Qinghai University, Xining 810016, China; lkqgsqy@126.com; 3 Sichuan Zoige Alpine Wetland Ecosystem National Observation and Research Station, Southwest Minzu University, Chengdu 610041, China

**Keywords:** oat (*Avena sativa*), nitrogen stress, Dof transcription factor, Root system architecture, cytokinin, protein interaction

## Abstract

Background: Oat (*Avena sativa* L.) is a vital forage and cereal crop widely cultivated on the Qinghai–Tibet Plateau, known for its outstanding adaptability to barren soils. Nitrogen (N) is a critical macronutrient, and the excessive application of N fertilizers has triggered severe ecological crises. Identifying key genes regulating adaptation to low N stress is essential for breeding N-efficient oat varieties. The DNA-binding with one finger (Dof) transcription factors play crucial roles in plant stress responses, but their precise molecular mechanisms in oat N-deficiency adaptation remain largely unknown. Results: In this study, we conducted a comprehensive genome-wide identification and unveiled 54 *AsDof* genes in the oat genome. Phylogenetic, synteny, and promoter analyses revealed their high evolutionary conservation and complex potential roles in stress networks. Transcriptome profiling under low N stress pinpointed *AsDof6.5* as a potential candidate. Ectopic expression of *AsDof6.5* in *Arabidopsis thaliana* significantly enhanced plant adaptation to low N. This was morphologically manifested as enhanced primary root elongation, increased root hair number, and significantly higher fresh weight under N-deprived conditions. Physiological assays further demonstrated that *AsDof6.5* overexpression efficiently reduced malondialdehyde (MDA) accumulation while simultaneously preserving soluble protein (SP) levels. Yeast two-hybrid (Y2H) and bimolecular fluorescence complementation (BiFC) assays demonstrated that AsDof6.5 has the potential to physically interact with the transcription factor AsWRKY3 and the cytokinin dehydrogenase AsCKX2 in the nucleus. Furthermore, *AsDof6.5* overexpression caused a significant transcriptional suppression of downstream *CKX2* homologs, which likely serves to fine tune local cytokinin homeostasis, thereby sustaining root developmental plasticity and vitality under severe low N stress. Conclusions: Our findings reveal a novel protein complex transcriptional regulatory hypothetical module. This module effectively enhances adaptation to low N by integrating nutrient starvation signaling with cytokinin homeostasis, providing a theoretical basis and valuable genetic resources for breeding N-efficient crops.

## 1. Background

Oat (*Avena sativa* L.) is a widely distributed annual herbaceous crop used for both grain and forage [1]. It is characterized by its extraordinary tolerance to barrenness, salinity, drought, and cold, making it a dominant crop in ecologically fragile areas, especially on the Qinghai–Tibet Plateau [2,3]. In recent years, the rapid growth of fertilizer application in China, which accounts for 30% of global nitrogen (N) fertilizer use, has significantly exceeded the world average [4,5]. The excessive use of N fertilizers has not synchronously increased grain yield; instead, it has led to frequent crop lodging and triggered severe water ecological and environmental safety issues due to severe N pollution [6,7,8]. Therefore, identifying low N-tolerant germplasm resources and excavating key genes regulating adaptation to low N are of profound significance for breeding new oat varieties, sustainably reducing chemical fertilizer usage, and protecting the fragile agricultural ecosystem [9,10].

N is an indispensable macronutrient for plant growth, development, and ultimate yield. In nutrient-barren soils, plants have evolved a high degree of morphological and physiological plasticity to actively adapt to low N stress. A classic and highly efficient morphological response is the significant remodeling of the root system architecture (RSA), such as stimulating rapid primary root elongation and robust root hair proliferation, to aggressively expand the absorption area for deep soil N. This complex developmental foraging strategy is tightly and dynamically regulated by endogenous phytohormones. Cytokinin (CK), acting as a paramount local and long-distance N signaling molecule, plays a central role in coordinating N availability with root growth. Typically, cytokinin oxidase/dehydrogenase (CKX) maintains CK homeostasis by irreversibly degrading CK. Increasing evidence suggests that under severe low N stress, plants intervene in endogenous CK concentrations by precisely downregulating the expression of *CKX* genes, thereby optimizing root foraging strategies to survive starvation.

Transcription factors act as key molecular switches in converting nutrient deprivation signals into morphogenetic responses. DNA-binding with one finger (Dof) proteins are plant-specific transcription factors widely recognized for their involvement in carbon/N metabolism and hormone signaling. For instance, the rice OsDOF11 coordinates carbon and N balance, robustly promoting root development and N absorption under low N conditions [11]. Overexpression of *dof1* in *Arabidopsis* resulted in superior growth, completely bypassing typical N-deficiency phenotypes [12]. Similarly, soybean *Gmdof1.4* has been shown to drastically improve N use efficiency (NUE) [13], while Dof5 strongly affects carbon and N metabolism in poplar [14]. Besides the Dof family, WRKY transcription factors constitute another core regulatory hub for plant responses to abiotic stresses. Interestingly, transcription factors frequently do not act alone but form sophisticated transcriptional complexes via direct protein–protein interactions (PPI) to achieve highly specific and amplified regulation of downstream target genes. However, whether Dof and WRKY proteins can physically interact in the nucleus to co-target hormone-degrading enzymes (such as CKX) to mediate crop adaptation to low N remains an unexplored territory in gramineous crops, especially in the genetically complex allohexaploid oat.

In this study, we conducted a comprehensive genome-wide identification of the oat *AsDof* gene family and deeply characterized their evolutionary features. Based on precise transcriptome profiling, *AsDof6.5* was identified as a prominently induced gene during N starvation. In this study, we aimed to identify key regulators of adaptation to low N in oat. Through genome-wide identification and transcriptome profiling, *AsDof6.5* was identified as a primary responsive candidate. By ectopically overexpressing *AsDof6.5* in *Arabidopsis*, we evaluated its biological function in root developmental plasticity under N starvation. Furthermore, by screening its interacting partners, we aimed to explore the potential molecular basis underlying *AsDof6.5*-mediated stress adaptation, providing theoretical insights for oat genetic improvement.

## 2. Materials and Methods

### 2.1. Plant Materials and Growth Conditions

Two oat varieties, the distinctively low N-tolerant ‘Jiayan 2’ and the low N-sensitive’ Qingyongjiu 016’ [9], were used for gene cloning.

Oat seedlings were grown hydroponically in a basal N-free modified Hoagland nutrient solution (Hopebio, Qingdao, China; Product No. HB8870-9). The exact ionic composition of this modified basal medium is detailed in Table 1. For the varying nitrogen treatments, the basal medium was supplemented with NH_4_NO_3_ to reach normal N (10 mM NH_4_NO_3_), low N (1.25 mM NH_4_NO_3_), or left unsupplemented for completely N-free (0 mM NH_4_NO_3_) conditions. Root and leaf samples were collected 15 days post-treatment for physiological and transcriptomic analyses. *Arabidopsis thaliana* (Columbia-0) was used for ectopic genetic transformation. Plants were grown in a controlled growth chamber at 22 °C with a 16 h/8 h light/dark cycle, 60% humidity, and a photosynthetic photon flux density (PPFD) of approximately 75 μmol·m^−2^·s^−1^. Root systems were scanned using the MSHOT MSX2-C scanner (Guangzhou Mingmei Optoelectronic Technology Co., Ltd., Guangzhou, China), and their morphological parameters were subsequently analyzed.

### 2.2. Genome-Wide Identification and Bioinformatics Analysis

The highly complex oat genome data were obtained from the GrainGenes database. The hidden Markov model (HMM) profile of the unique Dof domain (PF03106) was utilized to rigorously identify AsDof proteins using HMMER. Candidate sequences were manually validated using NCBI Batch CD-Search and SMART databases to prevent false positives. Physiochemical properties were analyzed using ExPASy ProtParam, and subcellular localizations were predicted using precise online tools. Multiple sequence alignments were performed using ClustalW2.1, and a neighbor-joining (NJ) phylogenetic tree was constructed using MEGA 7.0 with 1000 bootstrap replicates for high confidence. Conserved motifs were mapped via MEME. Chromosomal locations and crucial synteny analyses within oat and between *Arabidopsis*, rice, and wheat were conducted and visualized using Tbtools (version 1.098693). Cis-acting regulatory elements in the 2000 bp upstream promoter regions were predicted using Plant CARE.

### 2.3. Vector Construction and Arabidopsis Transformation

The full-length coding sequence (CDS) of *AsDof6.5* was amplified from the cDNA of the low N-tolerant variety ‘Jiayan 2’ and subsequently cloned into the pEGOEPubi vector. The recombinant overexpression plasmid was introduced into *Agrobacterium tumefaciens* strain GV3101 and successfully transformed into *Arabidopsis* via the floral dip method. Transgenic lines were screened on selective media, and positive T3 homozygous lines were identified by PCR and RT-qPCR for subsequent experiments. All primers used for cloning and vector construction are completely listed in Supplementary Table S1.

### 2.4. Adaptation to Low N Evaluation in Transgenic Arabidopsis

Seeds of wild-type (WT) and T3 transgenic lines (OE-3, OE-6, OE-16) were surface-sterilized and sown in square Petri dishes (10 × 10 cm) containing modified 1/2 MS agar medium with varying N concentrations. To ensure the complete removal of basal nitrogen while maintaining potassium homeostasis, standard 1/2 MS medium was modified by entirely omitting NH_4_NO_3_ and equimolarly replacing KNO_3_ with KCl. For the different N treatments, precise amounts of NH_4_NO_3_ were supplemented into this N-free basal medium to achieve final N concentrations of 10 mM NH_4_NO_3_ (acting as the high-N reference treatment, N:P ratio = 32:1), 3 mM (N:P ratio = 9.6:1), 0.3 mM (N:P ratio = 0.96:1), 0.15 mM (N:P ratio = 0.48:1), and 0 mM NH_4_NO_3_ (N:P ratio = 0:1). Seedlings were grown vertically to meticulously observe root system architecture alterations. Root length and plant fresh weight were recorded (*n* = 15 seedlings per genotype per treatment). For adult stage evaluation, seedlings were transferred to vermiculite-filled pots and constantly irrigated with modified Hoagland solutions at different N levels. The completely N-free (0 mM N) treatment was prepared using a commercial N-free Modified Hoagland Nutrient Solution mix (Hopebio, Qingdao, China; Product No. HB8870-9). The detailed molar concentrations of the N-free basal nutrient solution are provided in Table 1. For other N treatments, the required amounts of NH_4_NO_3_ were supplemented into this basal N-free medium.

The physiological parameters, including MDA content and SP content, were strictly measured using commercial assay kits (Comin Biotechnology, Suzhou, China) according to the manufacturer’s instructions.

### 2.5. Yeast Two-Hybrid (Y2h) Assay

The coding sequences of *AsDof6.5* and candidate interacting proteins (*AsWRKY3*, *AsWRKY51*, *AsDof5.10*, *AsCKX2*, *AsCKX6*) were cloned into the pGADT7 (AD) and pGBKT7 (BK) vectors, respectively. The recombinant plasmids were co-transformed into the yeast strain AH109 using established high-efficiency protocols [15]. Co-transformants were robustly selected on double dropout medium (SD/-Trp-Leu), and actual protein–protein interactions were stringently tested on triple (SD/-Trp-Leu-His) and quadruple (SD/-Trp-Leu-His-Ade) dropout media to completely rule out false-positive self-activation.

### 2.6. Bimolecular Fluorescence Complementation (BiFC) Assay

For the in vivo BiFC assay, the CDS of *AsDof6.5* was fused to the N-terminal fragment of YFP (P2YN), while candidate genes were fused to the C-terminal fragment of YFP (P2YC). The target constructs were mobilized into *Agrobacterium tumefaciens* EHA105 and subsequently co-infiltrated into 4-week-old healthy *Nicotiana benthamiana* leaves, following previously established and validated methodologies for assessing protein interaction complexes [16]. After 48–72 h of incubation in the dark, the reconstituted YFP fluorescence signals were observed and captured using a high-resolution confocal laser scanning microscope.

### 2.7. RNA Extraction and RT-qPCR

Total RNA was extracted from whole root and leaf tissues using the RNAprep Pure Plant Kit, and superior-quality first-strand cDNA was synthesized using the HiScript II Q RT SuperMix (Vazyme Biotech Co., Ltd., Nanjing, China). RT-qPCR was performed using the ChamQ Universal SYBR qPCR Master Mix on a Real-Time PCR System. The oat *AsActin* and *Arabidopsis AtActin2* genes were used as highly stable internal references for accurate normalization. Relative expression levels were rigorously calculated using the 2^−ΔΔCt^ method [17]. Corresponding primers are comprehensively listed in Supplementary Table S1.

#### 2.7.1. RNA Extraction, Library Construction, and Transcriptome Sequencing

Total RNA was extracted from whole root and leaf samples of ‘Jiayan 2’ and ‘Qingyongjiu 016’ under varying N treatments using a Plant RNA Extraction Kit (TaKaRa Bio Inc., Shiga, Japan). RNA purity and concentration were assessed using an IMPLEN spectrophotometer (IMPLEN, Westlake Village, CA, USA). RNA integrity was rigorously evaluated utilizing 1.5% agarose gel electrophoresis and the Agilent 2100 Bioanalyzer (Agilent Technologies, Santa Clara, CA, USA). For RNA-seq analysis, leaf and root samples were collected from three independent biological replicates per treatment. A total of 36 libraries were constructed and sequenced on the Illumina NovaSeq 6000 platform (Illumina, San Diego, CA, USA). The RNA sequencing and library construction were conducted by Novogene (Beijing, China). Sequencing libraries were generated and subsequently sequenced on an Illumina NovaSeq 6000 platform to generate paired-end reads. To ensure data reliability, raw reads were cleaned by removing adapter-containing sequences and low-quality reads (reads with >50% bases having a Q-value ≤ 20) to obtain high-quality clean reads. The resulting clean reads were then mapped to the *Avena sativa* reference genome (PepsiCo Oat OT3098 v2) for subsequent analysis. The raw RNA-seq data were submitted to the NCBI Sequence Read Archive (SRA) under the accession number PRJNA855044.

#### 2.7.2. Differential Expression Analysis and Expression Profiling

Gene expression levels were quantified as fragments per kilobase of transcript per million mapped reads (FPKM). The differential expression analysis between varying N treatments was performed utilizing the DEGseq R package (version 2.13). The resulting *p*-values were adjusted using the Benjamini and Hochberg’s approach to control the false discovery rate. Genes with an adjusted *p*-value (*padj*) < 0.05 and |log2 (FoldChange)| ≥ 1.0 were defined as significantly differentially expressed genes (DEGs). To identify crucial candidate genes responding to N starvation, the FPKM expression profile of the identified *AsDof* gene family members was extracted and visualized as a hierarchical clustering heatmap.

### 2.8. Statistical Analysis

All physiological, morphological, and transcriptomic experiments were conducted with at least three independent biological replicates. For phenotypic analyses, 15 individual seedlings (*n* = 15) were measured per genotype per treatment. Data were processed using Microsoft Excel and visualized with Origin 2026. Statistical significance was determined using one-way ANOVA followed by LSD multiple comparisons (*p* < 0.05).

## 3. Results

### 3.1. Identification and Structural Characterization of the Asdof Gene Family in Oat

**Figure 1:**
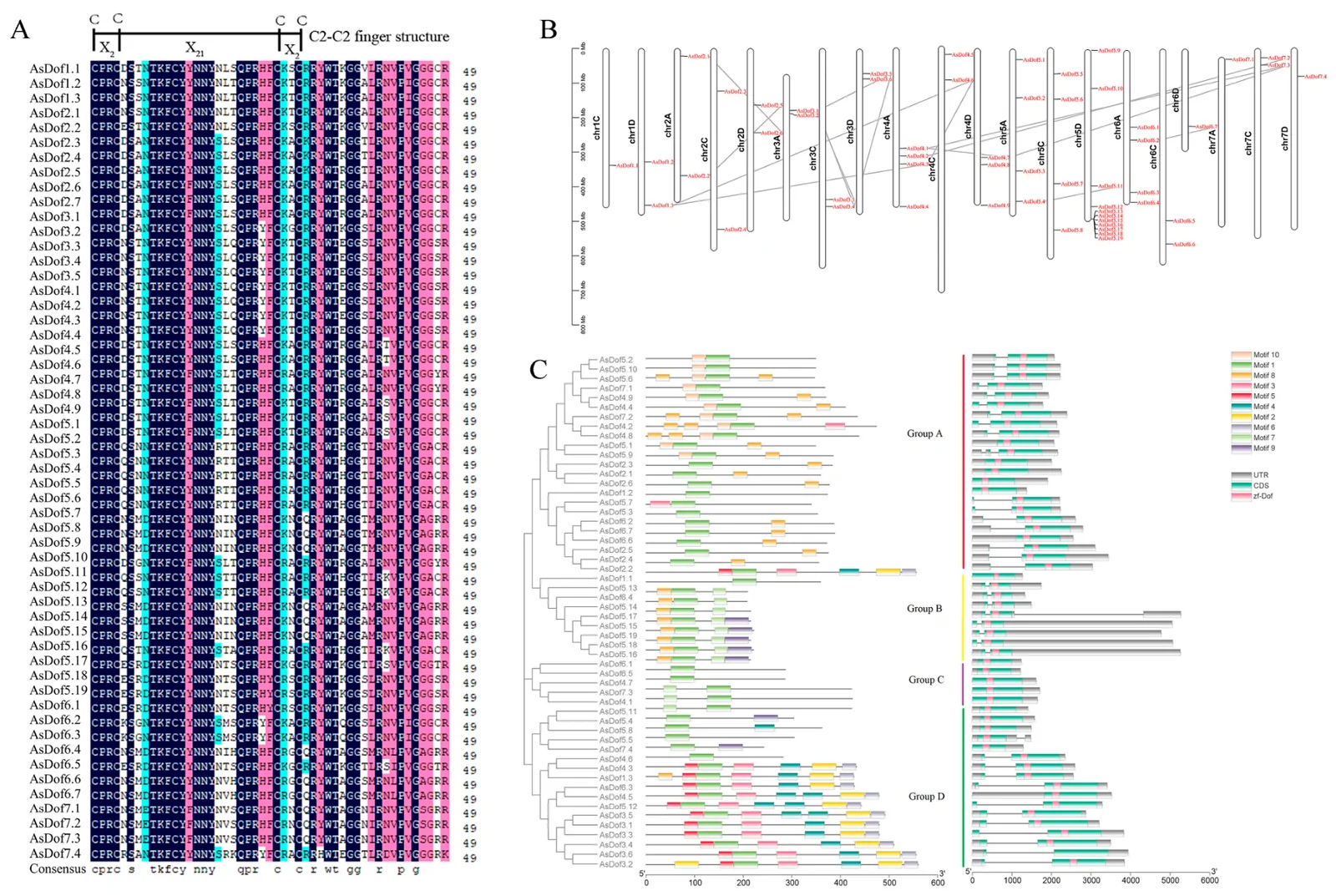
Identification, chromosomal localization, and structural characterization of the *AsDof* gene family in oat. (**A**) Multiple sequence alignment of the highly conserved zf-Dof domains (C_2_C_2_ zinc-finger structure) across the identified AsDof proteins. (**B**) Chromosomal mapping of the 54 *AsDofs* mapped onto the A, C, and D subgenomes of oat. (**C**) Comprehensive visualization of the unrooted phylogenetic tree (left), gene exon-intron structures (middle), and conserved MEME motifs (right) among different groups of AsDof proteins.

Leveraging the newly available high-quality oat genome, an exhaustive genome-wide search successfully identified 54 *AsDof* gene family members, designated as *AsDof1.1* to *AsDof7.4* based on their exact chromosomal mapping (Table 2). Intriguingly, multiple sequence alignment revealed an astonishingly high conservation of the C_2_C_2_-type zinc-finger structure (CX_2_C-X_21_-CX_2_C) containing exactly 49 amino acid residues at the N-terminus across all AsDof proteins (Figure 1A). This extreme structural rigidness within the zinc-finger domain underscores its indispensable role in mediating precise DNA-binding events throughout oat evolution. Subcellular localization prediction robustly indicated that all 54 AsDof proteins are strictly localized to the nucleus, perfectly aligning with their presumed function as master transcriptional regulators. Furthermore, comprehensive chromosomal mapping visualized a dynamic evolutionary landscape, revealing an uneven distribution across the 20 chromosomes within the A, C, and D subgenomes (17, 15, and 22 members, respectively) (Figure 1B). Gene structure and conserved motif analyses (Figure 1C) further corroborated their precise phylogenetic classification, with genes in the same group exhibiting highly synchronized exon-intron architectures and motif patterns, suggesting strong functional redundancies and conservation within specific clades.

### 3.2. Evolutionary Dynamics, Promoter Profiling, and Synteny Reveal Stress-Responsive Architectures

**Figure 2:**
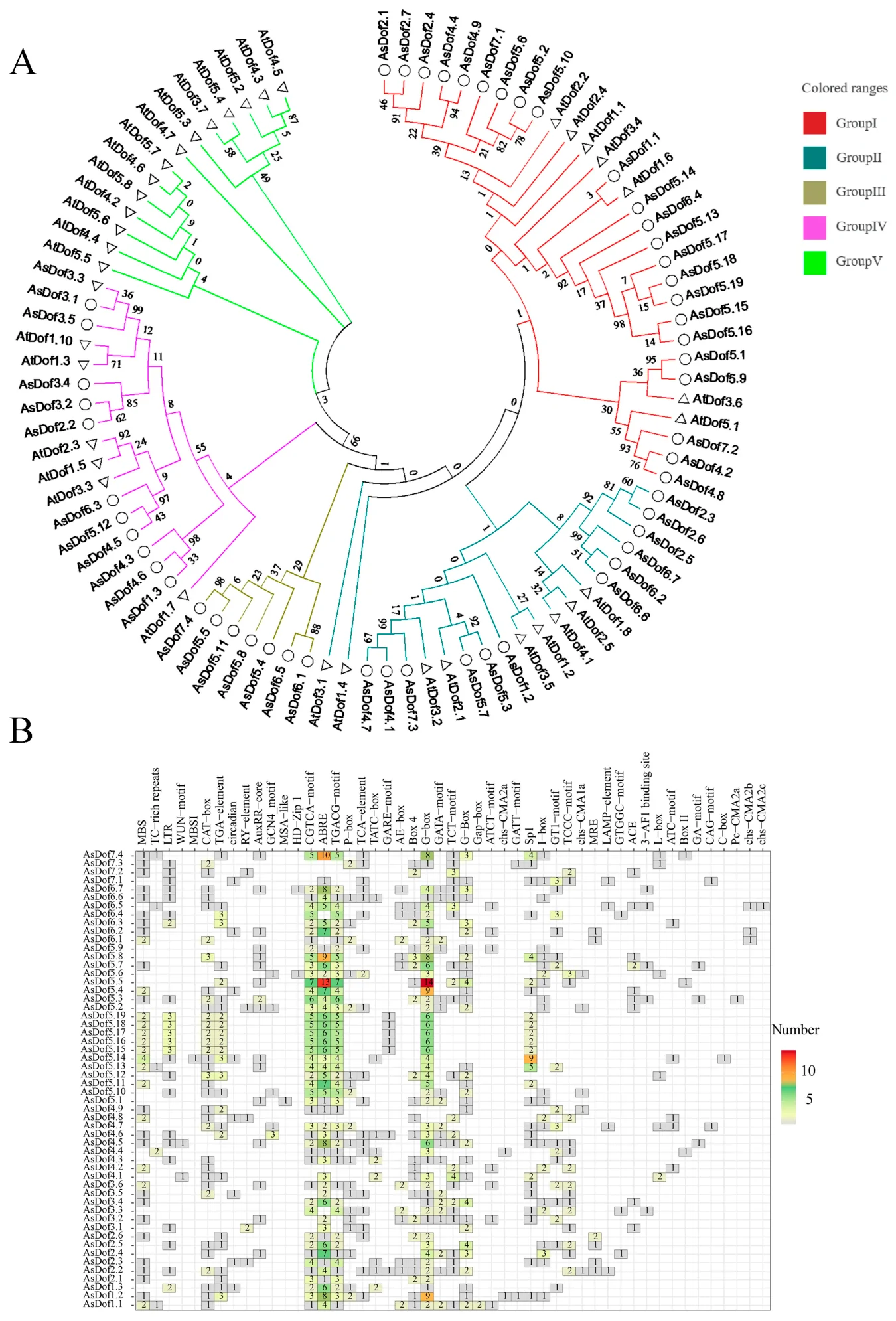
Phylogenetic and promoter regulatory network analysis of *AsDof* genes. (**A**) Unrooted phylogenetic tree showcasing the evolutionary relationship between 54 oat AsDof proteins (indicated by circles) and 36 *Arabidopsis* AtDof proteins (indicated by triangles). The five major groups are distinguished by different colors. (**B**) Heatmap predicting the number and distribution of diverse cis-acting regulatory elements in the 2000 bp upstream promoter regions of the *AsDof* genes.

To unravel the evolutionary trajectory of these genes, an unrooted phylogenetic tree incorporating the 54 AsDofs and 36 *Arabidopsis* AtDof proteins was constructed (Figure 2A). They clustered into five distinct major groups (Groups I to V). Remarkably, Group III exclusively contained seven AsDof members without any *Arabidopsis* counterparts, heavily implying a unique and specialized evolutionary divergence specifically tailored to the environmental adaptation of oat. To decode their potential regulatory networks, we scrutinized the 2000 bp upstream promoter regions (Figure 2B). The analysis uncovered an overwhelming abundance of cis-acting regulatory elements dedicated to severe abiotic and biotic stress responses, prominently including abscisic acid (ABRE), jasmonic acid (TGACG-motif), drought-inducible MYB binding sites (MBS), and defense/stress-responsive elements (TC-rich repeats). The dense accumulation of these elements strongly dictates that *AsDof* genes are deeply wired into the plant’s central stress response hub.

**Figure 3:**
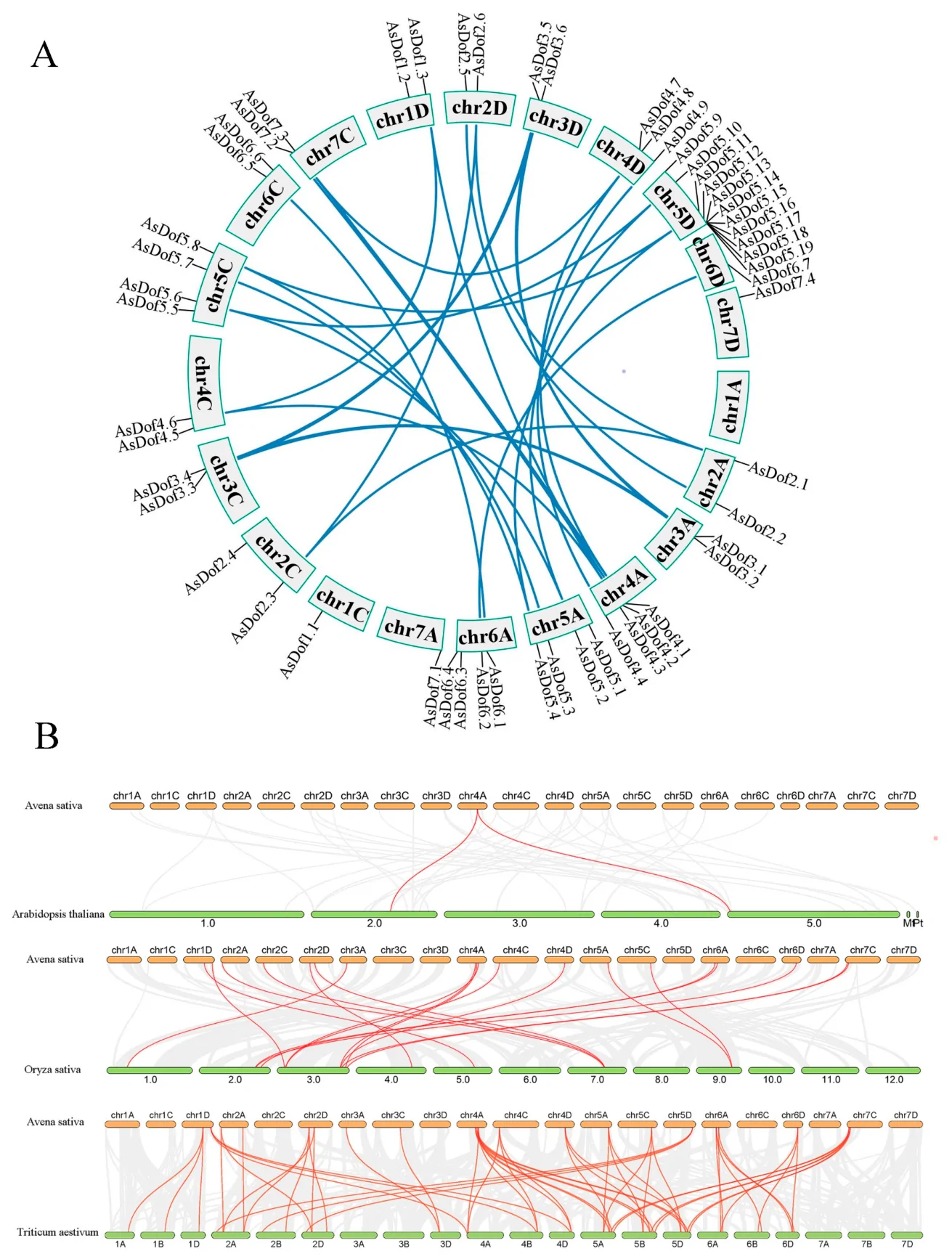
Genome-wide synteny analysis of *AsDof* genes. (**A**) Intraspecies synteny analysis displaying robust segmental duplication events of *AsDof* genes within the oat genome. Blue lines connect homologous gene pairs. (**B**) Interspecies synteny analysis illustrating the evolutionary collinearity between oat and three representative plant species: *Arabidopsis thaliana* (dicot), *Oryza sativa* (rice, monocot), and *Triticum aestivum* (wheat, monocot). Red lines highlight syntenic gene pairs.

Moreover, genome synteny analysis illuminated 27 pairs of robust segmental duplication events involving 33 *AsDof* genes within the oat genome (Figure 3A), highlighting duplication as the primary driving force for the expansion of this crucial gene family. Strikingly, interspecies synteny analysis revealed that oat share an extensive and highly significant collinearity with other stress-resilient gramineous crops (rice and wheat), but surprisingly negligible collinearity with the dicot model *Arabidopsis* (Figure 3B). This significant divergence strongly indicates that the Dof regulatory network has undergone distinct, grass-specific evolutionary pressures to maximize survival in challenging terrains.

### 3.3. Transcriptome Profiling Unmasks Asdof6.5 as a Low N-Responsive Regulator

**Figure 4:**
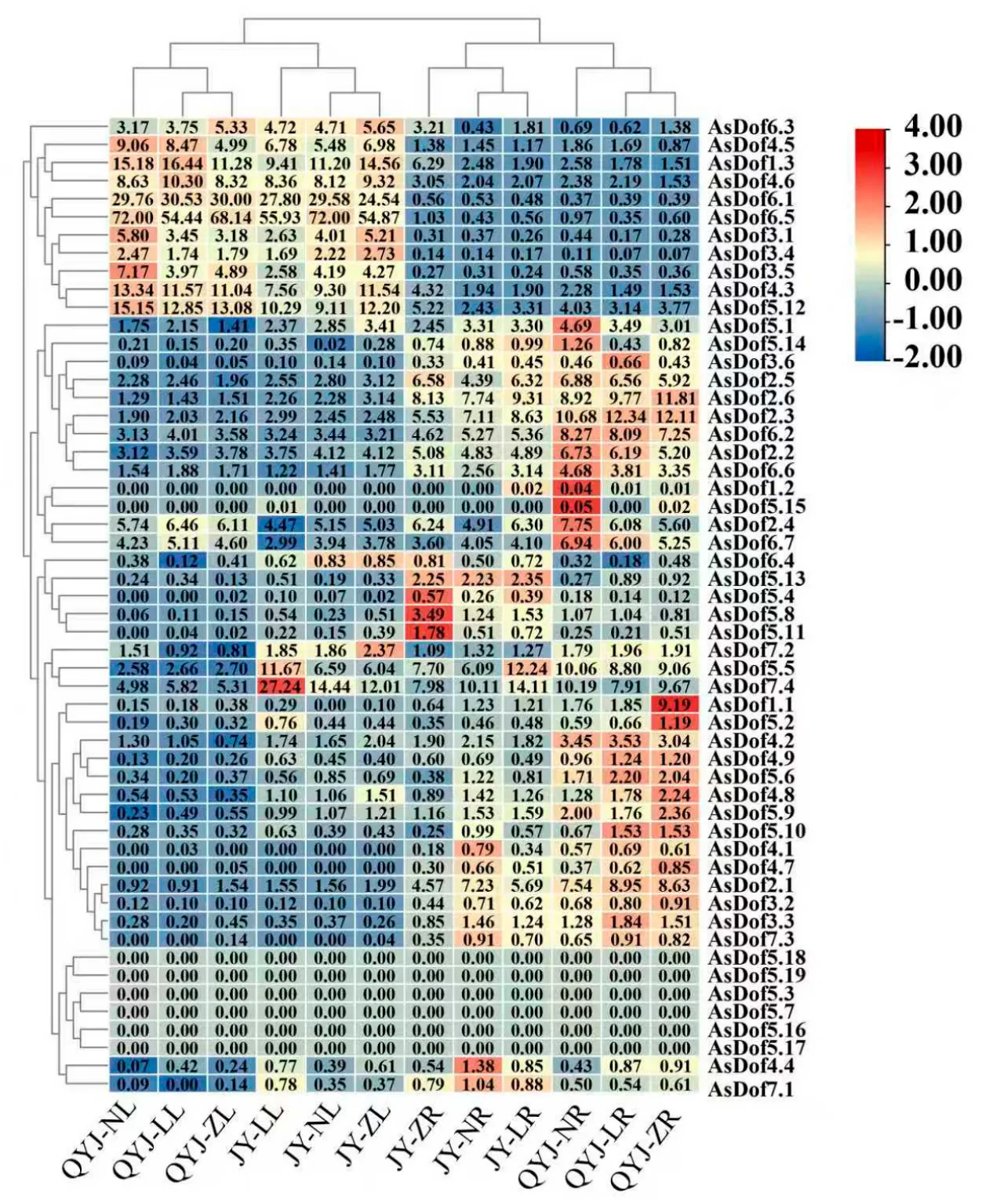
Expression profiling of *AsDof* genes under low N stress. Hierarchical clustering heatmap illustrating the relative expression patterns (FPKM values) of *AsDof* family members based on RNA-seq data (PRJNA855044) derived from oat leaves under Normal N, Low N, and N-free conditions. Red color indicates higher relative expression, while blue indicates lower relative expression. The highly responsive candidate, *AsDof6.5*, is marked for downstream functional analysis. Note: JY-NL stands for leaves of ‘Jiayan 2’ under10 mM NH_4_NO_3_, JY-LL stands for leaves of ‘Jiayan 2’ under 1.25 mM NH_4_NO_3_, JY-ZL stands for leaves of ‘Jiayan 2’ under 0 mM NH_4_NO_3_, QYJ-NL stands for leaves of ‘Qingyongjiu 016’ under 10 mM NH_4_NO_3_, QYJ-LL stands for leaves of‘Qingyongjiu 016’ under 1.25 mM NH_4_NO_3_, QYJ-ZL stands for leaves of ‘Qingyongjiu 016’ under 0 mM NH_4_NO_3_, JY-NR stands for roots of ‘Jiayan 2’ under 10 mM NH_4_NO_3_, JY-LR stands for roots of ‘Jiayan 2’ under 1.25 mM NH_4_NO_3_, JY-ZR stands for roots of ‘Jiayan 2’ under 0 mM NH_4_NO_3_, QYJ-NR stands for roots of ‘Qingyongjiu 016’ under 10 mM NH_4_NO_3_, QYJ-LR stands for roots of ‘Qingyongjiu 016’ under 1.25 mM NH_4_NO_3_, QYJ-ZR stands for roots of ‘Qingyongjiu 016’ under 0 mM NH_4_NO_3_.

To identify which *AsDof* genes are associated with long-term adaptation to N starvation, RNA-seq data from the low-N-tolerant (‘Jiayan 2’) and sensitive (‘Qingyongjiu 016’) varieties were analyzed. Quality control of the sequencing data showed that all samples had >96% clean reads, Q30 scores above 94%, and stable GC content, confirming the high data quality and its suitability for accurate gene quantification and differential expression analysis (Supplementary Table S2). The hierarchical clustering heatmap (Figure 4) illustrated distinct, condition-specific expression shifts among the family members. *AsDof6.5* exhibited a highly responsive expression pattern, identifying it as a primary candidate for further functional analysis. Its expression was strongly induced under low N treatments, with FPKM values peaking at 72. To further validate this transcriptomic pattern, we performed RT-qPCR analysis to examine the relative expression of *AsDof6.5* across varying N levels (Supplementary Figure S1). The results confirmed that *AsDof6.5* expression was significantly upregulated under low N (1.25 mM) and N-free (0 mM) conditions. Notably, the tolerant variety ‘Jiayan 2’ exhibited a much stronger and more rapid induction of *AsDof6.5* compared to the sensitive variety ‘Qingyongjiu 016’. This consistent and robust expression pattern at both the transcriptomic and transcriptional levels firmly positions *AsDof6.5* as a primary functional candidate for orchestrating the oat low N adaptation network.

### 3.4. Ectopic Overexpression of Asdof6.5 Significantly Enhances Adaptation to Low N and Root Plasticity

**Figure 5:**
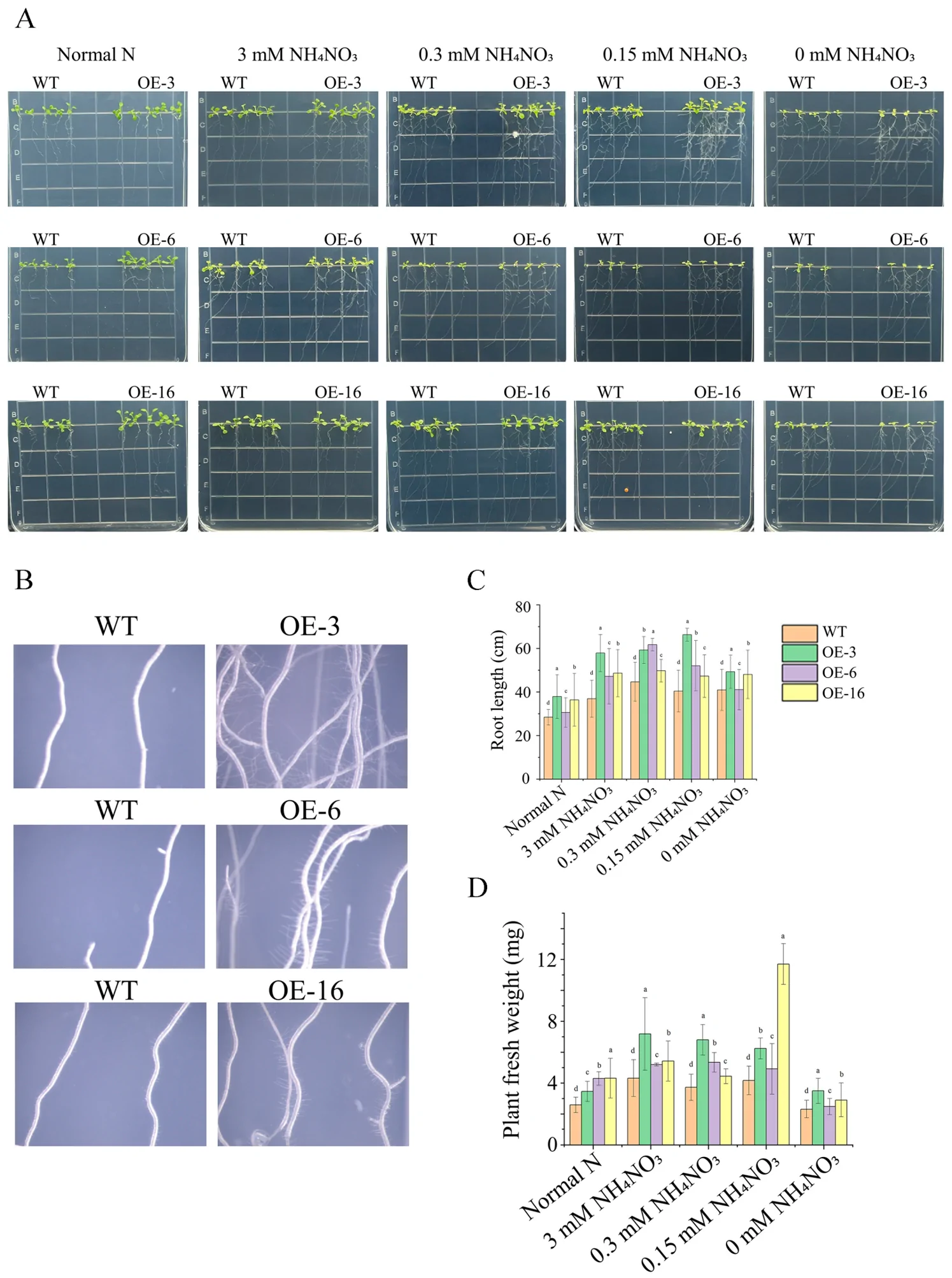
Ectopic overexpression of *AsDof6.5* enhances adaptation to low N and root remodeling in *Arabidopsis* seedlings. (**A**) Visual growth phenotypes of wild-type (WT) and three independent *AsDof6.5* transgenic lines (OE-3, OE-6, OE-16) grown vertically on 1/2 MS media containing varying N concentrations (Normal N, 3 mM, 0.3 mM, 0.15 mM, and 0 mM NH_4_NO_3_). (**B**) Representative root systems captured by the MSHOT MSX2-C scanner. (**C**) Quantification of primary root length. Different lowercase letters above the bars indicate highly significant differences at *p* < 0.01 (one-way ANOVA), the same as below. (**D**) Quantification of whole plant fresh weight. Error bars represent the standard deviation (SD) of three independent biological replicates. Different lowercase letters above the bars indicate statistically significant differences at *p* < 0.05, as determined by one-way ANOVA followed by LSD multiple comparisons.

To experimentally validate the commanding role of this gene, *AsDof6.5* was ectopically overexpressed in *Arabidopsis*. Twenty independent T3 homozygous overexpression lines (OE) were successfully generated, with RT-qPCR confirming extraordinarily high transcript abundance. Lines OE-3, OE-6, and OE-16 were judiciously selected for downstream stress challenges (Supplementary Figure S2). Under normal N conditions on 1/2 MS plates, OE lines and wild-type (WT) plants displayed comparable baseline growth. However, when subjected to stringent N-deprivation (ranging from 3 mM down to completely 0 mM NH_4_NO_3_), the OE lines unleashed a vigorous and highly plastic root system response. They exhibited dramatically promoted primary root elongation (Figure 5C) alongside a strong and robust proliferation of root hairs (Figure 5A,B) compared to the stunted WT roots. This phenomenal root expansion represents a classic and highly efficient ‘foraging’ strategy. Consistently, the entire plant fresh weight of the OE lines was remarkably maintained, vastly outperforming the fragile WT under low N stress (Figure 5D).

**Figure 6:**
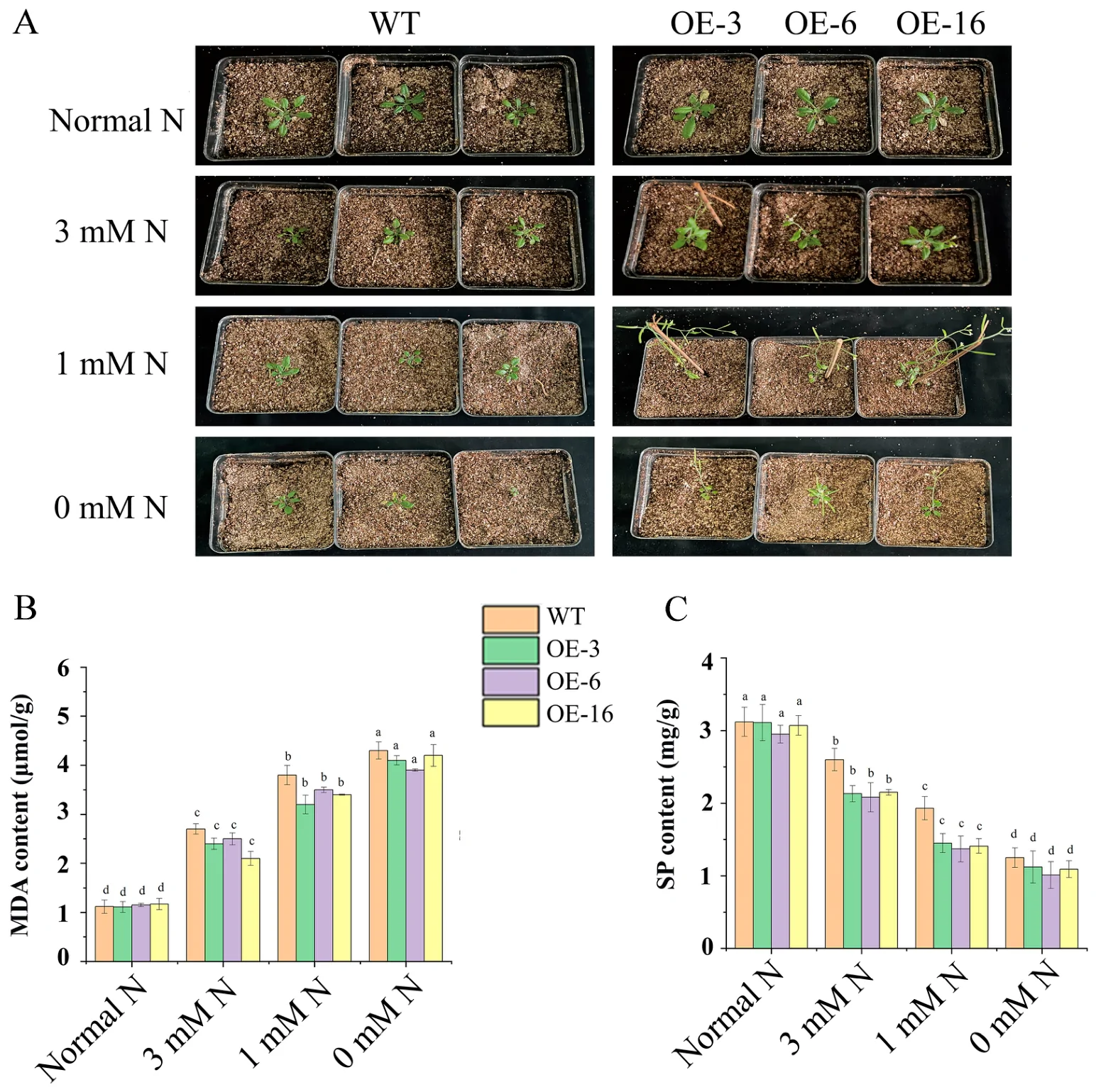
Physiological evaluation of transgenic *Arabidopsis* plants under long-term low N stress. (**A**) Phenotypes of WT and transgenic plants (OE-3, OE-6, OE-16) cultivated in vermiculite soil for 30 days under different N irrigation treatments (Normal N, 3 mM, 1 mM, and 0 mM N). (**B**) MDA content, serving as an indicator of membrane lipid peroxidation and oxidative damage. (**C**) SP content, reflecting the N assimilation efficiency and cellular vitality. Error bars represent the standard deviation (SD) of three independent biological replicates. Different lowercase letters above the bars indicate statistically significant differences at *p* < 0.05, as determined by one-way ANOVA followed by LSD multiple comparisons.

Pushing this to the adult stage, plants were challenged with long-term severe N deprivation in soil pots (Figure 6A). N starvation inevitably triggers devastating oxidative bursts. Measuring physiological hallmarks of stress revealed that while MDA contents surged as N concentration dropped, the destructive magnitude of MDA accumulation was significantly and strictly suppressed in the OE lines compared to the WT (Figure 6B). Conversely, while soluble protein (SP) contents naturally collapsed under starvation, the OE lines miraculously maintained significantly higher SP levels, averting cellular breakdown (Figure 6C). Collectively, these potent morphological and physiological data definitively prove that *AsDof6.5* acts as a powerful enhancer, actively shielding the plant from oxidative destruction while increasingly promoting root foraging capabilities to dominate low N environments.

### 3.5. AsDof6.5 Physically Interacts with AsWRKY3 and AsCKX2

To explore the molecular network associated with *AsDof6.5*, we predicted potential protein–protein interaction partners using the STRING database. Five candidates (AsDof5.10, AsWRKY3, AsWRKY51, AsCKX2, and AsCKX6) were selected for experimental validation.

**Figure 7:**
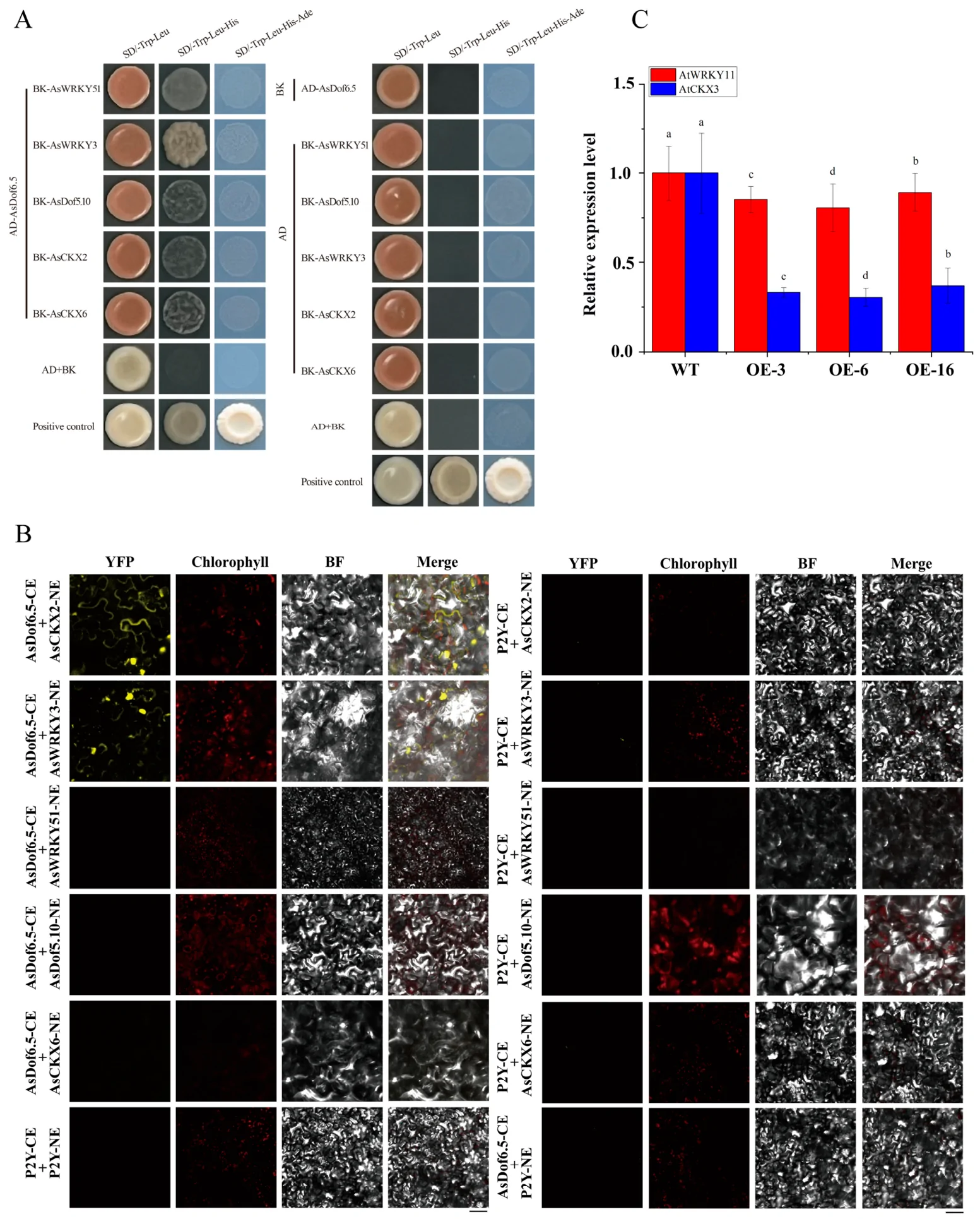
*AsDof6.5* forms a regulatory complex with AsWRKY3 and AsCKX2 to suppress downstream target genes. (**A**) Yeast two-hybrid (Y2H) assay detecting the direct physical interactions of AsDof6.5 with candidate proteins. BK-53+AD-T7 was the positive control and BK-lam+AD-T7 was the negative control. (**B**) Bimolecular fluorescence complementation (BiFC) assay verifying the in vivo interactions of AsDof6.5 with AsWRKY3 and AsCKX2 in *Nicotiana benthamiana* epidermal cells. Strong Yellow Fluorescent Protein (YFP) signals were primarily detected in the nucleus. Combinations with empty vectors (e.g., P2Y-CE + P2Y-NE) served as negative controls and showed no background fluorescence. The scale bar represents 50 µm. (**C**) RT-qPCR analysis revealing the significant transcriptional suppression of *AtWRKY11* and *AtCKX3* in *AsDof6.5* overexpressing *Arabidopsis* lines compared to WT. Error bars represent the standard deviation (SD) of three independent biological replicates. Different lowercase letters above the bars indicate statistically significant differences at *p* < 0.05, as determined by one-way ANOVA followed by LSD multiple comparisons.

Y2H assays showed that yeast co-transformants expressing AsDof6.5 with AsWRKY3 or AsCKX2 could grow on the triple dropout medium (SD/-Trp-Leu-His). However, growth on the more stringent quadruple dropout medium (SD/-Trp-Leu-His-Ade) was weak or barely detectable (Figure 7A), suggesting that their physical interactions might be relatively weak or transient in the yeast system.

Therefore, five candidate proteins (AsDof5.10, AsWRKY3, AsWRKY51, AsCKX2, and AsCKX6) were selected for bimolecular fluorescence complementation (BiFC) assays to further verify their in vivo interactions (Figure 7B). To further investigate these physical interactions in vivo, we performed BiFC assays in *Nicotiana benthamiana* leaves. Strong YFP fluorescence signals were observed in the nucleus of cells co-infiltrated with AsDof6.5-NE/AsCKX2-CE and AsDof6.5-NE/AsWRKY3-CE, suggesting that these proteins can form physical modules within plant cells (Figure 7B). Crucially, no YFP fluorescence was detected in the negative control group co-infiltrated with the empty vectors (P2Y-CE + P2Y-NE), confirming the specificity of the observed fluorescent signals in living plant cells.

Given that CKX enzymes are key regulators of cytokinin (CK) degradation, we subsequently examined whether *AsDof6.5* influences the expression of CK-related responsive genes. RT-qPCR analysis of the *AsDof6.5*-overexpressing *Arabidopsis* lines revealed that the transcript levels of *AtWRKY11* and the cytokinin dehydrogenase gene *AtCKX3* were significantly downregulated compared to the WT (Figure 7C). While direct binding evidence is required to define these as primary targets, these results suggest that *AsDof6.5*, possibly through its interaction with AsWRKY3 and AsCKX2, modulates a downstream signaling cascade that leads to the transcriptional suppression of *CKX* homologs. This coordinated regulation—potentially involving both transcriptional modulation and physical interaction with the CK degradation machinery—may contribute to the maintenance of cytokinin homeostasis and enhanced root development under N starvation.

## 4. Discussion

Oat inevitably encounters a barrage of severe abiotic stresses during its life cycle, leading to devastating yield losses [18,19,20]. Transcription factors play the most pivotal roles in decoding stress signals and driving evolutionary adaptation [21]. The Dof family, distinguished by its highly conserved C_2_C_2_ zinc-finger motif at the N-terminus, is uniquely renowned for highly specific DNA-binding capabilities [21,22,23,24,25]. While the first identified Dof factor, ZmDof1 from maize, was initially tied to light regulation [26], our understanding has vastly expanded. The cultivated oat genome (AACCDD) is notoriously complex [3]. Our comprehensive genome-wide analysis identified 54 *AsDof* members with an uneven subgenomic distribution, pointing towards potential homologous gene loss accompanied by extensive segmental and tandem duplications that drove the family’s expansion. Exon-intron structures, serving as steadfast fingerprints of evolutionary relationships [27], perfectly mirrored our phylogenetic clades, cementing their functional conservation [28]. For instance, our group V aligns closely with *Arabidopsis AtDof4.7* [29], strongly hinting at shared, fundamental biological functions transcending species.

*Cis*-acting elements are crucial molecular switches controlling dynamic responses to environmental stimuli [30]. The abundant presence of ABRE, TGACG, and MBS elements in the *AsDof* promoters facilitates their integration into complex stress-responsive networks. For example, corresponding stress-responsive elements in *TaAIDFa* have been shown to significantly improve drought and osmotic tolerance in transgenic wheat [31]. Our high-depth RNA-seq analysis (Figure 4) clearly demonstrated that multiple *AsDof* genes are highly responsive to low N stress, elevating them as promising candidates for N-deficiency genetic engineering.

Rapid root morphological remodeling is the most direct and crucial adaptive strategy for plants desperately foraging in N-deficient soils [32]. In our study, ectopic overexpression of *AsDof6.5* in *Arabidopsis* yielded a spectacular phenotype: significantly elongated primary roots, increased root hair density, and strikingly elevated biomass under severe low N conditions. This perfectly aligns with landmark findings on rice *OsDOF11* [33] and maize *ZmDof1* [34], conclusively validating the highly conserved and dominant function of Dofs in nutrient foraging among staple crops. Beyond mere morphology, severe low N stress invariably triggers lethal reactive oxygen species (ROS) bursts and lipid peroxidation. Our compelling physiological data proved that *AsDof6.5* overexpression acted as an effective biochemical shield, dramatically reducing MDA accumulation while fiercely protecting soluble protein levels from degradation. This proves that AsDof6.5 is not merely a structural architect but a powerful metabolic guardian, physiologically armoring the plant to sustain high N-assimilation efficiency amidst severe environmental starvation.

Dof proteins are fascinating due to their dual-functional binding activities, seamlessly interacting with both target DNA sequences and crucial companion proteins to orchestrate highly complex transcriptional machineries [35,36,37,38]. A key finding of our study is the elucidation of the downstream molecular network mediated by *AsDof6.5*. Utilizing both Y2H and BiFC assays, we discovered that AsDof6.5 directly and physically docks with the transcription factor AsWRKY3 and the key cytokinin-degrading enzyme AsCKX2.

**Figure 8:**
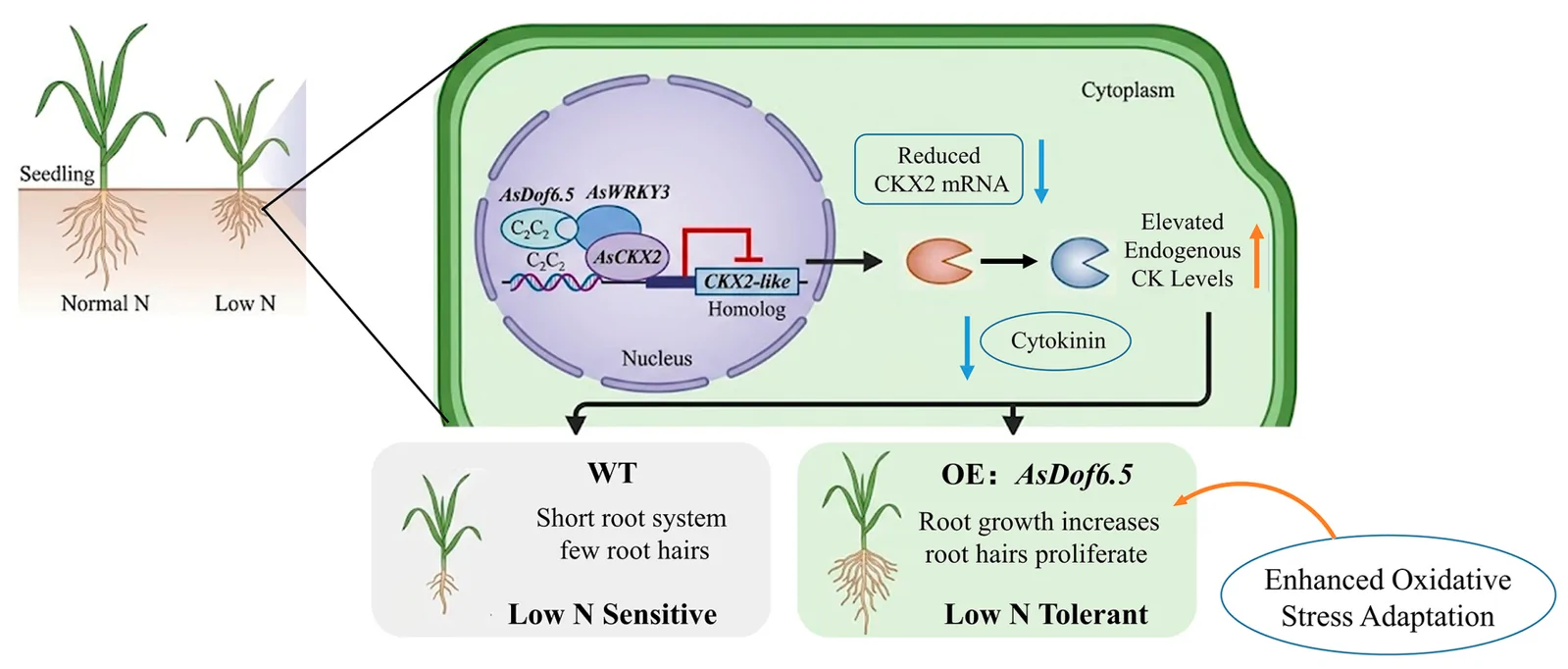
Hypothetical working model of *AsDof6.5*-mediated low N adaptation in epidermal cells. Under low N starvation, AsDof6.5 physically docks with AsWRKY3 and AsCKX2 in the nucleus. This complex is hypothesized to suppress the expression of downstream CKX2-like homologs, while the direct interaction with AsCKX2 may potentially restrict CK degradation. The inferred maintenance of endogenous CK promotes increased root hair proliferation and primary root elongation, ultimately armoring the plant against oxidative damage and severe low N stress.

By integrating the physical interaction data with our transcriptomic findings, we propose a hypothetical dual-level regulatory model for *AsDof6.5*-mediated adaptation to low N (Figure 8). Upon perception of altered nutrient availability (such as absolute N deficiency and the subsequently shifted N:P ratio), *AsDof6.5* exhibits a putative two-pronged strategy to preserve endogenous cytokinin (CK). At the transcriptional level, *AsDof6.5* interacts with the transcription factor *AsWRKY3* in the nucleus. Although direct binding to target promoters requires further validation via chromatin immunoprecipitation (ChIP) assays, we hypothesize that this regulatory complex may target and suppress the promoters of downstream *CKX* homologs, thereby reducing the abundance of *CKX* transcripts. Concurrently, at the post-translational level, the direct physical interaction between *AsDof6.5* and the cytokinin dehydrogenase *AsCKX2* suggests that *AsDof6.5* may potentially modulate the stability or enzymatic activity of *AsCKX2*. While we did not directly quantify endogenous CK concentrations or *AsCKX2* enzyme kinetics in this study, the observed transcriptional downregulation implies that this synergistic suppression might curtail CK degradation. Consequently, the inferred maintenance of a local CK pool is likely essential for sustaining root apical meristem activity and promoting root hair proliferation under nutritional stress.

In classical root developmental biology, maintaining appropriate CK gradients is essential for sustaining rapid cell division, root meristematic activity, and root hair outgrowth [39]. The *AsDof6.5*-mediated suppression of *CKX2* expression, therefore, represents an effective molecular adaptation strategy: the plant actively restricts cytokinin degradation to promote continuous root growth and foraging within N-deficient environments. Compared to conventional single-factor models, this Dof-WRKY synergistic repression of hormone-degrading enzymes offers a novel perspective on how gramineous crops integrate nutritional deficit signaling with sophisticated phytohormone networks to achieve enhanced abiotic stress adaptation.

It is important to acknowledge the inherent limitations of the experimental approaches used in this study. First, our transcriptomic and RT-qPCR analyses were conducted using bulk whole-root or whole-leaf tissues at 15 days post-treatment. Therefore, the observed gene expression patterns represent a long-term developmental adaptation rather than an immediate-early stress response [40]. Furthermore, bulk tissue analysis averages the transcriptional profiles across highly heterogeneous cell types (e.g., epidermis, cortex, and vasculature). Consequently, it remains challenging to distinguish whether the observed transcriptional downregulation of CKX homologs in the transgenic lines is strictly due to reduced per-cell expression, or partially a secondary result of altered cell population ratios (e.g., increased proliferation of root hair cells lacking CKX expression). Future studies incorporating single-cell RNA sequencing (scRNA-seq) or spatial transcriptomics will be crucial to definitively uncouple cell-type-specific transcriptional responses from organ-level morphological adaptations.

While the ectopic expression in *Arabidopsis* clearly demonstrates the conserved function of *AsDof6.5* in enhancing adaptation to low N, future work focusing on the generation of *AsDof6.5* overexpression and knockout lines in oat will be essential to fully elucidate its role and explore its breeding potential in this specific allohexaploid crop.

## 5. Conclusions

In this study, a comprehensive array of 54 *AsDof* gene family members was rigorously identified at the genome-wide level in oat. We definitively pinpointed *AsDof6.5* as a gene exceptionally responsive to severe low N stress. Ectopic expression of *AsDof6.5* in *Arabidopsis thaliana* significantly and positively regulated the entire plant’s tolerance to extreme N deficiency. Under low N starvation, the overexpressing lines exhibited significantly enhanced root system development (marked by promoted primary root elongation and increased root hair density), higher biomass retention, and improved oxidative stress adaptation (showcasing significantly decreased MDA and well-preserved SP levels). Further deep mechanistic investigations revealed that the AsDof6.5 protein forms a direct, physical transcriptional regulatory complex with AsWRKY3 and AsCKX2 in the nucleus. Crucially, we propose that this complex potentially suppresses the expression of downstream CKX2 homologous genes, thereby putatively restricting cytokinin (CK) degradation to maintain growth-stimulating endogenous CK levels. This hypothesized hormonal intervention may ultimately drive aggressive root foraging and fortify oxidative adaptation under low N stress. Our research successfully outlines a putative mechanism of AsDof6.5-mediated adaptation to low N, providing significant candidate genes and theoretical blueprints for the future breeding of elite, highly N-efficient oat varieties.

## Figures and Tables

**Table 1:** Detailed ionic composition of the basal N-free modified nutrient solution.

Component	Chemical Formula	Concentration
Potassium chloride	KCl	2.05 mM
Dipotassium phosphate	K_2_HPO_4_·3H_2_O	2.00 mM
Magnesium sulfate	MgSO_4_·7H_2_O	1.00 mM
Iron (III) sodium EDTA	EDTA-FeNa	30.4 µM
Boric acid	H_3_BO_3_	25.0 µM
Manganese sulfate	MnSO_4_·H_2_O	2.0 µM
Copper sulfate	CuSO_4_	0.5 µM
Zinc sulfate	ZnSO_4_·H_2_O	2.0 µM
Sodium molybdate	Na_2_MoO_4_	0.5 µM

**Table 2:** Information about the *AsDof* gene family in oats.

Gene Name	Gene ID	*Arabidopsis thaliana* ID	Chromosome Localization	Protein Structural Domain Location	Amino Acid Length	Isoelectric Point	Relative Molecular Mass (kD)
*AsDof1.1*	AVESA.00001b.r3.1Cg0001244	*AtDof1.1*	1C	183–221	359	10.4	36.88
*AsDof1.2*	AVESA.00001b.r3.1Dg0001582	*AtDof3.5*	1D	79–134	373	5.79	39.62
*AsDof1.3*	AVESA.00001b.r3.1Dg0003266	*AtDof3.3*	1D	101–156	429	7.51	46.39
*AsDof2.1*	AVESA.00001b.r3.2Ag0000129	*AtDof2.2*	2A	52–108	359	7.68	35.76
*AsDof2.2*	AVESA.00001b.r3.2Ag0001817	*AtDof3.7*	2A	85–134	383	8.65	39.07
*AsDof2.3*	AVESA.00001b.r3.2Cg0000736	*AtDof5.3*	2C	47–103	355	7.22	35.35
*AsDof2.4*	AVESA.00001b.r3.2Cg0002519	*AtDof2.5*	2C	77–126	375	8.65	38.44
*AsDof2.5*	AVESA.00001b.r3.2Dg0001742	*AtDof3.7*	2D	83–132	377	8.65	38.42
*AsDof2.6*	AVESA.00001b.r3.2Dg0002695	*AtDof2.2*	2D	44–100	355	7.78	35.51
*AsDof3.1*	AVESA.00001b.r3.3Ag0001032	*AtDof5.2*	3A	175–231	556	5.12	60.17
*AsDof3.2*	AVESA.00001b.r3.3Ag0001117	*AtDof5.2*	3A	104–160	480	7.23	51.71
*AsDof3.3*	AVESA.00001b.r3.3Cg0001661	*AtDof3.3*	3C	137–193	510	8.79	54.84
*AsDof3.4*	AVESA.00001b.r3.3Cg0001745	*AtDof5.2*	3C	178–234	560	5.06	60.96
*AsDof3.5*	AVESA.00001b.r3.3Dg0000593	*AtDof5.2*	3D	175–231	556	5.01	60.15
*AsDof3.6*	AVESA.00001b.r3.3Dg0000687	*AtDof5.2*	3D	104–160	480	8.12	51.94
*AsDof4.1*	AVESA.00001b.r3.4Ag0001607	*AtDof5.4*	4A	122–176	423	9.12	44.20
*AsDof4.2*	AVESA.00001b.r3.4Ag0001942	*AtDof5.1*	4A	172–221	474	9.84	49.57
*AsDof4.3*	AVESA.00001b.r3.4Ag0002279	*AtDof3.3*	4A	100–155	428	7.9	46.29
*AsDof4.4*	AVESA.00001b.r3.4Ag0003866	*AtDof3.6*	4A	144–200	410	8.1	41.99
*AsDof4.5*	AVESA.00001b.r3.4Cg0000307	*AtDof5.2*	4C	69–125	443	6.33	47.00
*AsDof4.6*	AVESA.00001b.r3.4Cg0001261	*AtDof3.3*	4C	105–160	433	7.9	46.99
*AsDof4.7*	AVESA.00001b.r3.4Dg0001955	*AtDof5.4*	4D	122–176	423	9.04	44.19
*AsDof4.8*	AVESA.00001b.r3.4Dg0002304	*AtDof5.1*	4D	136–185	438	9.22	45.07
*AsDof4.9*	AVESA.00001b.r3.4Dg0003930	*AtDof3.6*	4D	107–163	370	8.86	38.07
*AsDof5.1*	AVESA.00001b.r3.5Ag0000405	*AtDof3.6*	5A	53–108	349	9.72	36.21
*AsDof5.2*	AVESA.00001b.r3.5Ag0001252	*AtDof3.6*	5A	121–176	349	8.91	35.98
*AsDof5.3*	AVESA.00001b.r3.5Ag0001989	*AtDof1.2*	5A	60–115	353	8.41	36.84
*AsDof5.4*	AVESA.00001b.r3.5Ag0002716	*AtDof1.2*	5A	38–85	362	4.67	36.48
*AsDof5.5*	AVESA.00001b.r3.5Cg0000386	*AtDof1.3*	5C	50–104	242	6.17	25.69
*AsDof5.6*	AVESA.00001b.r3.5Cg0000662	*AtDof3.6*	5C	121–176	347	8.91	35.86
*AsDof5.7*	AVESA.00001b.r3.5Cg0001544	*AtDof1.3*	5C	50–105	340	8.61	35.63
*AsDof5.8*	AVESA.00001b.r3.5Cg0002337	*AtDof1.3*	5C	39–86	305	4.53	30.91
*AsDof5.9*	AVESA.00001b.r3.5Dg0000020	*AtDof5.1*	5D	92–147	385	9.36	40.05
*AsDof5.10*	AVESA.00001b.r3.5Dg0000828	*AtDof3.6*	5D	121–176	349	8.91	35.98
*AsDof5.11*	AVESA.00001b.r3.5Dg0002242	*AtDof1.3*	5D	41–88	304	4.51	30.99
*AsDof5.12*	AVESA.00001b.r3.5Dg0003010	*AtDof5.2*	5D	116–172	492	6.5	52.35
*AsDof5.13*	AVESA.00001b.r3.5Dg0003365	*AtDof3.4*	5D	50–107	209	8.23	22.19
*AsDof5.14*	AVESA.00001b.r3.5Dg0003366	*AtDof3.4*	5D	48–105	215	8.56	22.53
*AsDof5.15*	AVESA.00001b.r3.5Dg0003368	*AtDof3.4*	5D	57–114	222	8.84	23.33
*AsDof5.16*	AVESA.00001b.r3.5Dg0003369	*AtDof3.4*	5D	50–107	215	8.59	22.54
*AsDof5.17*	AVESA.00001b.r3.5Dg0003370	*AtDof3.4*	5D	50–107	215	8.59	22.54
*AsDof5.18*	AVESA.00001b.r3.5Dg0003372	*AtDof3.4*	5D	56–113	221	8.84	23.22
*AsDof5.19*	AVESA.00001b.r3.5Dg0003375	*AtDof3.4*	5D	50–107	215	8.59	22.54
*AsDof6.1*	AVESA.00001b.r3.6Ag0000975	*AtDof5.4*	6A	48–103	287	8.8	29.58
*AsDof6.2*	AVESA.00001b.r3.6Ag0001206	*AtDof2.5*	6A	78–133	387	8.82	39.44
*AsDof6.3*	AVESA.00001b.r3.6Ag0002913	*AtDof5.2*	6A	105–161	480	5.88	50.89
*AsDof6.4*	AVESA.00001b.r3.6Ag0003314	*AtDof3.4*	6A	55–112	208	8.23	21.70
*AsDof6.5*	AVESA.00001b.r3.6Cg0002918	*AtDof5.4*	6C	47–97	286	8.42	29.67
*AsDof6.6*	AVESA.00001b.r3.6Cg0003200	*AtDof2.5*	6C	60–115	372	8.66	38.19
*AsDof6.7*	AVESA.00001b.r3.6Dg0000887	*AtDof2.5*	6D	78–133	388	8.82	39.60
*AsDof7.1*	AVESA.00001b.r3.7Ag0000081	*AtDof3.6*	7A	101–157	368	8.62	38.04
*AsDof7.2*	AVESA.00001b.r3.7Cg0000322	*AtDof5.1*	7C	136–185	435	9.2	44.71
*AsDof7.3*	AVESA.00001b.r3.7Cg0000683	*AtDof5.4*	7C	123–177	425	9.09	44.30
*AsDof7.4*	AVESA.00001b.r3.7Dg0000837	*AtDof1.4*	7D	89–143	282	8.7	30.01

## Data Availability

The RNA-seq raw data supporting the findings of this study have been deposited in the NCBI Sequence Read Archive (SRA) under the accession number PRJNA855044.

## Supplementary Materials

The following supporting information can be downloaded at: https://www.mdpi.com/article/10.3390/genes17101248/s1, Figure S1: Relative expression levels of *AsDof6.5* in different oat varieties under varying nitrogen treatments; Figure S2: Validation of *AsDof6.5* relative expression levels in T3 homozygous transgenic Arabidopsis lines via RT-qPCR; Table S1: Primers used in this study; Table S2: Quality assessment of RNA-seq sequencing data from oat varieties under low N stress.

## Abbreviations

N	Nitrogen
NUE	Nitrogen use efficiency
CK	Cytokinin
CKX	Cytokinin oxidase/dehydrogenase
MDA	Malondialdehyde
SP	Soluble protein
Y2H	Yeast two-hybrid
BiFC	Bimolecular fluorescence complementation
RT-qPCR	Quantitative real-time PCR
