# Supplementary material for: Ectopic Expression of AsDof6.5 from Oat (Avena sativa L.) Enhances Low Nitrogen Tolerance in Transgenic Arabidopsis thaliana
Source: Genes (Basel). 2026 Oct 9;17(10):1248. doi: 10.3390/genes17101248 (PMC13704636; doi:10.3390/genes17101248)
Supplement: Supplementary file 1 [file genes-17-01248-s001.zip › Supplementary File.pdf]

Supplementary Information

Supplementary Table S1. Primers used in this study.

Primer Name	Sequence (5' to 3')
AsDof6.5 -F	ATGCAGGACTTCCAATCCATCC
AsDof6.5 -R	CTAGGGTAGGTAGACGGACGAGT
qPCR-*AsDof6.5*-F	5'-CACGGAAGCCAAGAACCAG-3'
qPCR-*AsDof6.5*-R	5'-GCGAAGTTGATGGTGTTGGA-3'
AsActin-F	5'-CCAGAGTCTAAGACGCAACC-3'
AsActin-R	5'-CGTGAAAGAATGACCCAAAT-3'
pEGOEPubi-*AsDof6.5* -F	ACTAGGGTCTCGCACCATGCAGGACTTCCAATCCATCC
pEGOEPubi-*AsDof6.5* -R	ACTAGGGTCTCTACCGCTAGGGTAGGTAGACGGACGAGT
UBI-F (Sequencing)	TTAGCCCTGCCTTCATACGC
GUS-R (Sequencing)	GGGTCCTAACCAAGAAAATGAA
qPCR-*AtWRKY11* -F	CGGTTCACTCCACTTCCTCT
qPCR-*AtWRKY11* -R	GACGGTGGAGGAACGATTTG
qPCR-*AtCKX3* -F	CTGCGTGGCTATGGATTGAG
qPCR-*AtCKX3* -R	CGTACCGAAACGTTTGTCCA
β-*actin2*-F	TGGGCCTACTGGTCTTACTACTG
β-*actin2*-R	ACATACCCACGCTTCAGATCCT
AsDof6.5-AD-F	GCCATGGAGGCCAGTGAATTCATGCAGGACTTCCAA
AsDof6.5-AD-R	ATTCATCTGCAGCTCGAGCTCCTAGGGTAGGTAGAC
AsWRKY3-BK-F	ATGGCCATGGAGGCCGAATTCATGATGACCATGGAT
AsWRKY3-BK-R	CCGCTGCAGGTCGACGGATCCTCAGAGCTTGTGCAG
AsWRKY51-BK-F	ATGGCCATGGAGGCCGAATTCATGATGACCATGGAT
AsWRKY51-BK-R	CCGCTGCAGGTCGACGGATCCCTAGTCCAACCTGCG

AsDof5.10-BK-F	ATGGCCATGGAGGCCGAATTCATGCTCTCCCACGTC
AsDof5.10-BK-R	CCGCTGCAGGTCGACGGATCCTCAGAACGGACGGTC
AsCKX6-BK-F	ATGGCCATGGAGGCCGAATTCATGGCCAGCTTCGTA
AsCKX6-BK-R	CCGCTGCAGGTCGACGGATCCTTATGTTGCTGCAAT
AsCKX2-BK-F	ATGGCCATGGAGGCCGAATTCATGCAGAGGATGGAG
AsCKX2-BK-R	CCGCTGCAGGTCGACGGATCCTCAAGCCAGTGGTGC
AD-F (Sequencing)	GGACGGACCAAACTGCGTA
BK-R (M13 Sequencing)	GAGCGGATAACAATTTCACAC
P2YN-*AsDof6.5* -F	CATTTACGAACGATAGTTAATTAAATGCAGGACTTCCAA
P2YN-*AsDof6.5* -R	CACTGCCACCTCCTCCACTAGTGGGTAGGTAGACGGA
P2YC-*AsWRKY3* -F	CATTTACGAACGATAGTTAATTAAATGATGACCATGGAT
P2YC-*AsWRKY3* -R	CACTGCCACCTCCTCCACTAGTGAGCTTGTGCAGCTC
P2YC-*AsWRKY51* -F	CATTTACGAACGATAGTTAATTAAATGATGACCATGGAT
P2YC-*AsWRKY51* -R	CACTGCCACCTCCTCCACTAGTGTCCAACCTGCGCTT
P2YC-*AsDof5.10* -F	CATTTACGAACGATAGTTAATTAAATGCTCTCCCACGTC
P2YC-*AsDof5.10* -R	CACTGCCACCTCCTCCACTAGTGAACGGACGGTCCGG
P2YC-*AsCKX6* -F	CATTTACGAACGATAGTTAATTAAATGGCCAGCTTCGTA
P2YC-*AsCKX6* -R	CACTGCCACCTCCTCCACTAGTTGTTGCTGCAATGTT
P2YC-*AsCKX2* -F	CATTTACGAACGATAGTTAATTAAATGCAGAGGATGGAG
P2YC-*AsCKX2* -R	CACTGCCACCTCCTCCACTAGTAGCCAGTGGTGCGTT
P2YC-R (Sequencing)	GCTCAGGTAGTGGTTGTCGG
P2YN-R (Sequencing)	TTGAAGTCGATGCCCTTCAG

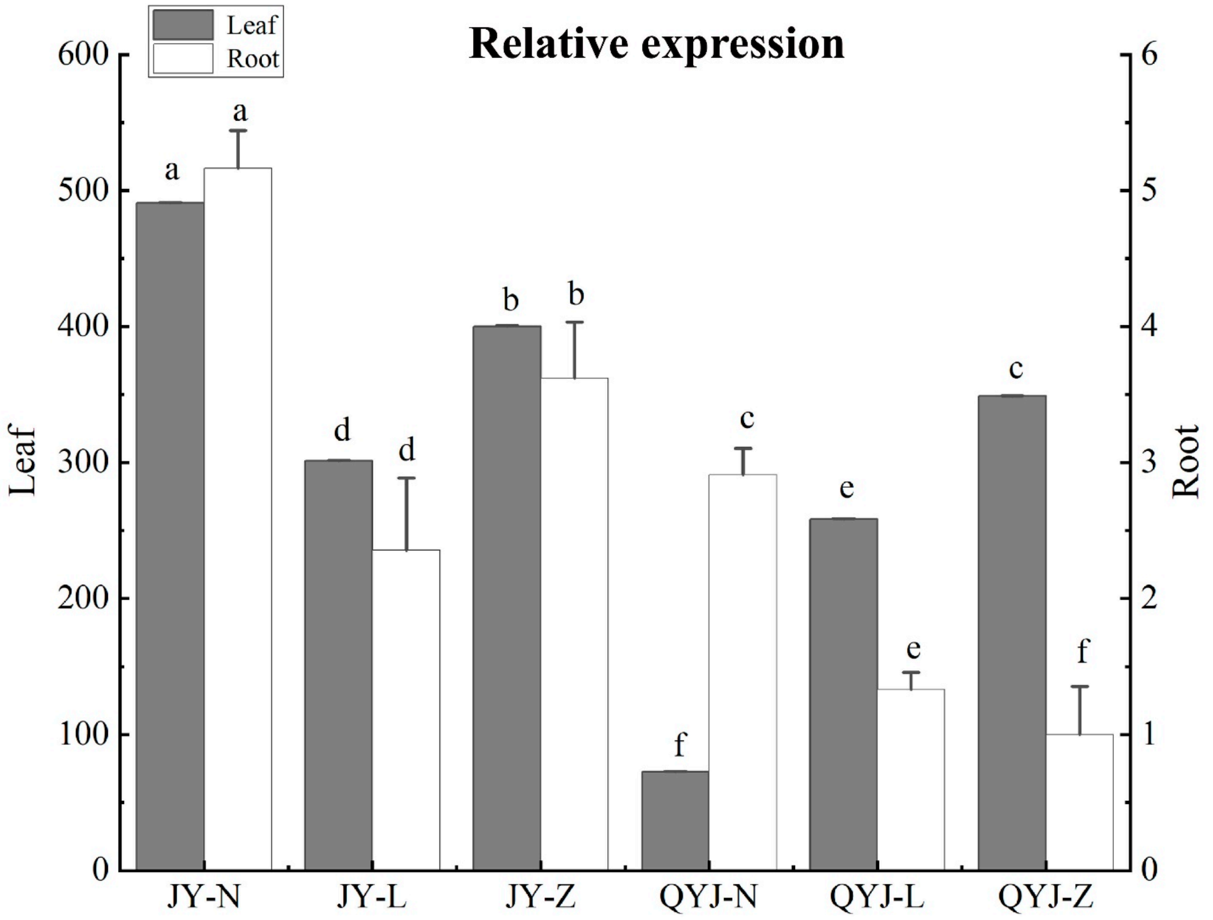

Supplementary Figure S1. Relative expression levels of *AsDof6.5* in different oat varieties under varying nitrogen treatments. Expression patterns of *AsDof6.5* were analyzed by RT-qPCR. 'JY' represents the low N-tolerant variety 'Jiayan 2', and 'QYJ' represents the low N-sensitive variety 'Qingyongjiu 016'. JY_N, JY_L, and JY_Z indicate the root and leaf samples of 'Jiayan 2' treated with 10 mM (Normal N), 1.25 mM (Low N), and 0 mM (N-free) NH_4NO_3, respectively. Similarly, QYJ_N, QYJ_L, and QYJ_Z indicate the root and leaf samples of 'Qingyongjiu 016' under 10 mM, 1.25 mM, and 0 mM NH_4NO_3 treatments. Data are presented as the mean ± SD of three biological replicates. Different lowercase letters above the bars indicate highly significant differences in the relative expression of *AsDof6.5* at $P < 0.01$ (one-way ANOVA).

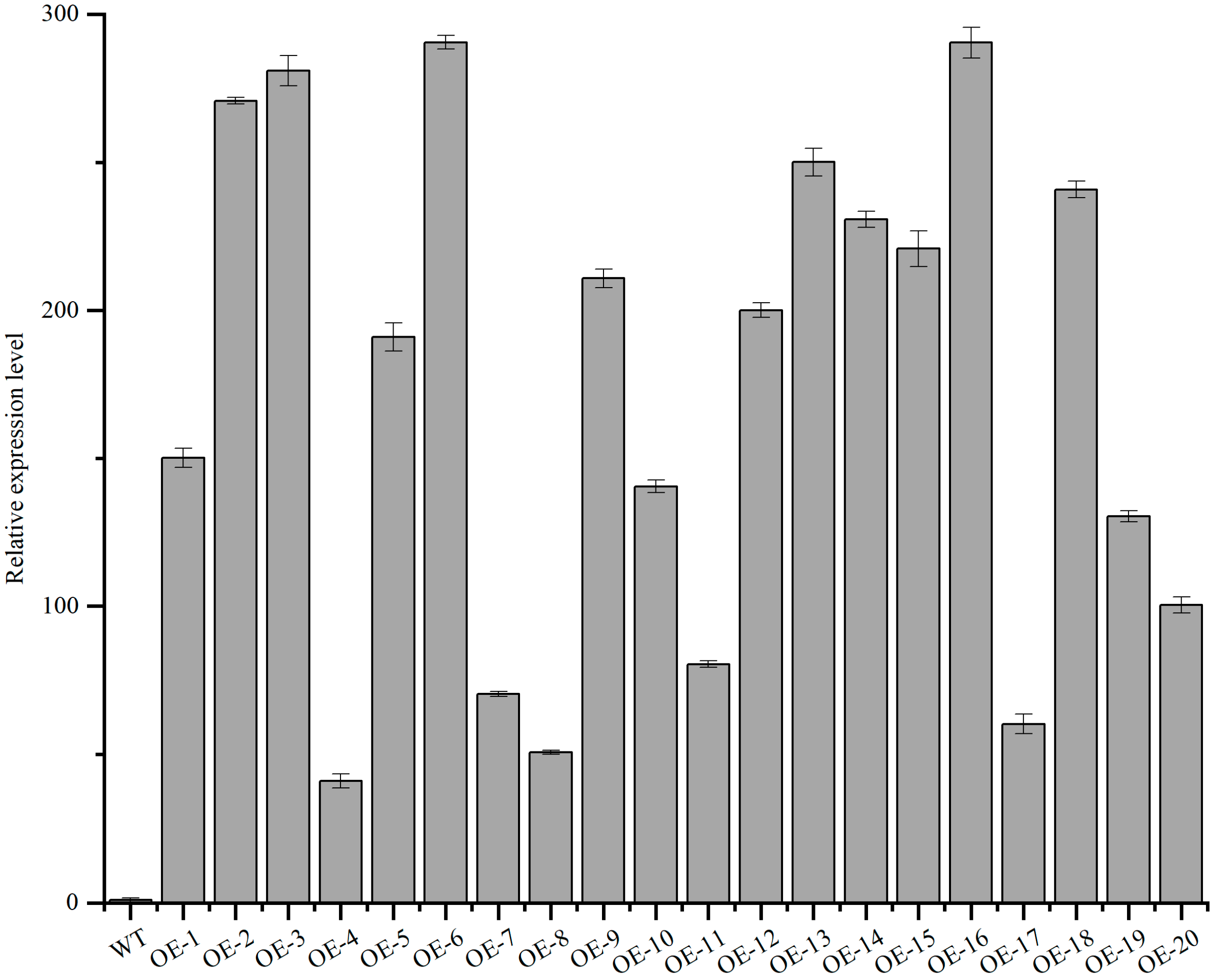

Supplementary Figure S2. Validation of *AsDof6.5* relative expression levels in T3 homozygous transgenic *Arabidopsis* lines via RT-qPCR.
